# Obesity-related measures and successful ageing among community-dwelling older adults in India: a cross-sectional study

**DOI:** 10.1038/s41598-022-21523-7

**Published:** 2022-10-13

**Authors:** T. Muhammad, Arun Balachandran, Pradeep Kumar, Shobhit Srivastava

**Affiliations:** 1 International Institute for Population Sciences, Mumbai, 400088 Maharashtra India; 2 University of Maryland, College Park, MD 20742 USA; 3 Population Council, New Delhi, India

**Keywords:** Geriatrics, Public health

## Abstract

Obesity is a chief lifestyle disease globally and causes a significant increase in morbidities. Overweight/ obesity prevalence has been rising faster in India compared to the world average. Therefore, the study examined the association between overweight/ obesity and successful ageing among older population in India. We also explored the gender difference in risks posed by obesity on successful ageing and the different socio-economic correlates associated with successful ageing. This study utilized data from India’s first nationally representative longitudinal ageing survey (LASI-2017-18). The effective sample size for the present study was 31,464 older adults with a mean age of 69.2 years (SD: 7.53). Overweight/ obesity was defined as having a body mass index of 25 or above. The study carried out a bivariate analysis to observe the association between dependent and independent variables. Further, multivariable analysis was conducted to examine the associations after controlling for individual socio-demographic, lifestyle and household/community-related factors. The study included 47.5% men and 52.5% women. It was found that the prevalence of obesity/overweight was higher among older women compared to older men (23.2% vs 15.5%). Similarly, high-risk waist circumference (32.7% vs 7.9%) and high-risk waist-hip ratio (69.2% vs 66.5%) were more prevalent among older women than older men. The study found significant gender differences (men-women: 8.7%) in the prevalence rate of successful ageing (p < 0.001). The chances of successful ageing were significantly higher among older adults who were not obese/overweight [AOR: 1.31; CI 1.31–1.55], had no high-risk waist circumference [AOR: 1.41; CI 1.29–1.54], and those who had no high-risk waist-hip ratio [AOR: 1.16; CI 1.09–1.24] compared to their respective counterparts. Interaction results revealed that older women who were not obese/overweight had a lower likelihood of successful ageing compared to the older men who was not obese/overweight [AOR: 0.86; CI 0.80–0.93]. Similarly, older womens who had no high-risk waist circumference [AOR: 0.86; CI 0.80–0.96] and no high risk-hip ratio [AOR: 0.81; CI 0.73–0.89] were less likely to have successful ageing compared to their counterparts, respectively. Being overweight/ obese and having high-risk waist circumference and waist-hip ratio were found to be significant factors associated with less successful ageing among older adults, especially women in this study. The current findings highlight the importance of understanding the modifiable factors, including nutritional awareness and developing targeted strategies for promoting successful ageing.

## Introduction

With the continuous decrease in fertility and increase in life expectancy, population ageing is expected to rise across the globe and is expected to be one of the principal social changes of the twenty-first century^1^. Population above age 60 is projected to be 3.1 billion by 2100, which is a triple fold increase from that of 2017^2^. While the relative share of the older population is higher in Western countries, the pace of population ageing and the absolute number of older adults are higher in Asian countries^3^. India has around 104 million population aged 60 and above, forming 8.6% of its population. The proportion is expected to rise to 20% of its population by 2050^4^.

While there has been an increase in life expectancy over time, it was recognized that a rise in years lived may not necessarily be ‘ideal’ if it leads to more years of unhealthy life or add burden to younger generations^5,6^. The multidimensional concept of successful ageing was formulated in the context to describe the quality of ageing^7^. The concept has developed beyond a biomedical approach into a wider understanding of social and psychological adaptation processes in later life. While there is no exclusive definition for the concept of successful ageing, several studies empirically estimate successful ageing markers^8^. Most of the operational definitions of successful ageing use physiological constructs such as physical functioning, well-being constructs (like life satisfaction) and engagement in social and productive work^9^. While population ageing has been on the rise across almost all parts of the world, the experience with successful ageing has been mixed. Measures aimed at quantifying successful ageing have found that the rate of successful ageing to be as diverse as 10.9% in Korea^10^ to around 30% in France^11^. A review of 28 studies examined several definitions and metrics of successful ageing, and found a proportion of 35.8% of the population as successfully ageing, with smoking and diabetes as significant correlates in addition to socio-economic predictors^12^.

Another aspect of the demographic change has been the rise in non-communicable and lifestyle diseases. Obesity is a chief lifestyle disease globally and causes a significant increase in morbidities^13^. Obesity has been a major issue for the last three decades in advanced regions such as the USA and Europe^14^. Obesity and overweight prevalence has been rising faster in India compared to the world average. The prevalence of overweight increased from 8.4 to 15.5% among women between 1998 and 2015, and the prevalence of obesity increased from 2.2 to 5.1% over the same period^15^. Obesity has direct association with disability status^16^, cognitive abilities^17^, mental health^18^, chronic conditions^19^ and multi-morbidities^20^ in older population. In addition, to an increase in morbidities, obesity also increases the likelihood of mortality and decreases the quality of life years^21^. In this backdrop, a holistic understanding of health and wellbeing of older adults calls for an analysis of the multidimensional concept of successful ageing in the context of rising obesity.

However, the risks posed by obesity on successful ageing in India has been unexplored in literature. Evidence from other parts of the world suggests significant associations between obesity/ overweight status and successful ageing, with variations across age groups. Obesity was negatively associated with successful ageing indicators among the French older adults between age 65 and 75^11^. The English Longitudinal Study of Ageing (ELSA) suggested that an excess BMI decreased healthy and disease-free life expectancy among older adults between ages 50–75^12^. Evidence from China suggests that men with obesity/overweight were more successful agers, and women with underweight had a negative relationship with indicators of successful ageing. At the same time, obesity status was not significantly associated with successful ageing indicators for both men and women above age 75 years^8^.

In this paper, we examined the association between overweight/ obesity and successful ageing among the older population in India, using the data from the Longitudinal Ageing Study in India (LASI). We also explored the gender difference in risks posed by obesity on successful ageing and the different socio-economic correlates associated with ageing successfully.

## Material and methods

### Study design and participants

The data for this study come from India's first nationally representative longitudinal ageing survey (LASI-2017-18, wave 1), which looks at the health, economic, and social drivers and effects of population ageing in India^22^. Except for Sikkim, the sample comprised 72,250 older adults aged 45 and above, as well as their spouses, from all Indian states and union territories. To choose the final units of observation, the LASI uses a multistage stratified area probability cluster sampling methodology. The last unit of observation was households with at least one person aged 45 or older. This research offers empirical information on demographics, household economic status, chronic health problems, symptom-based health conditions, functional and mental health, biomarkers, health care usage, job and employment, and more. It was created to examine the impact of altering policies and behavioural outcomes in India, and it allows for cross-state and cross-national studies of ageing, health, economic status, and social behaviours. The LASI wave-1 report contains detailed information about the sampling frame. For this investigation, the effective sample size was 31,464 older individuals aged 60 years or older^22^.

### Variable description

#### Outcome variable

Successful ageing differs from region to region, and the present paper defined successful ageing based on five criteria measured by utilizing self-reported survey questionnaires^23,24^. The five components were 1. Absence of chronic diseases 2. Freedom from disability 3. High cognitive ability 4. Free from depressive symptoms, and 5. Active social engagement in life. The older adults satisfying all the above conditions were considered as the successful ageing group^8^. The five components in detail are as follow:

1. Absence of chronic diseases: Chronic diseases were assessed from the self-report question of “Have you been diagnosed with conditions listed below by a doctor?” The diseases were hypertension, chronic heart diseases, stroke, any chronic lung disease, diabetes, cancer or malignant tumour, any bone/joint disease, any neurological/psychiatric disease or high cholesterol^25^. Respondents were classified as having no chronic diseases if they reported to have none of the above-mentioned diseases.
2. Freedom from disability: Activities of Daily Living (ADL) is a term used to refer to normal daily self-care activities (such as movement in bed, changing position from sitting to standing, feeding, bathing, dressing, grooming, personal hygiene etc.)^22^. The ability or inability to do ADLs is used to assess a person's functional state, particularly in the case of disabled people and older adults^22^. If respondents reported to be able to perform all of their daily activities independently, they were classed as having no disability^22^.
3. High cognitive ability: Five broad domains were used to assess cognitive ability (memory, orientation, arithmetic function, executive function and object naming)^22^. Memory was measured using immediate word recall, delayed word recall; orientation was measured using time and place measure; arithmetic function was measured through backward counting, serial seven and computation method; executive function was measured through paper folding and pentagon drawing method, and object naming was lastly done to measure the cognitive function among older adults^22^. Using the domain wise measure, a composite score of 0–43 was calculated. The bottom tenth percentile is used as a marker for poor cognitive performance^22^. The cognitive ability of older persons who did not fall into the lowest tenth percentile was regarded to be high^22^.
4. Free from depressive symptoms: The CIDI-SF (Short Form Composite International Diagnostic Interview) was used to calculate the likelihood of major depression in older persons with dysphoria with a score of 0-10^22^. This scale has been validated in field settings and is commonly used in population-based health studies to determine a probable mental diagnosis of major depression^22^. The cut-off of three was used for severe depression among older adults^22^. The older adults who scored less than three were considered free from depressive symptoms^22^.
5. Active social engagement in life: Respondents were considered to be socially engaged if they participate in the following activities. Eat out of house (Restaurant/Hotel); Go to park/beach for relaxing/entertainment; Play cards or indoor games; Play outdoor games/sports/exercise/jog/yoga; Visit relatives /friends; Attend cultural performances /shows/Cinema; Attend religious functions /events such as bhajan/satsang/prayer; Attend political/community/organization group meetings; Read books/newspapers/magazines; Watch television/listen radio and use a computer for e-mail/net surfing etc.^22^. Older adults who reported any of the above activities at least once in a month were considered socially active.

### Explanatory variables

#### Anthropometric measurement (key exposures)

Anthropometric measurements, such as height, weight, waist circumference, and hip circumference, were performed for all the LASI survey participants. The items "overweight" and "obesity" were classified as "no" and "yes". Obese/overweight was defined as having a body mass index of 25 or above^26^. No and yes were used to indicate high-risk waist circumference. Men and women who have High-risk waist circumferences were defined as those with circumferences of more than 102 cm and 88 cm, respectively^27^. No and yes were used to indicate the high-risk waist-hip ratio. Men and women with waist-hip ratios more than 0.90 cm and 0.85 cm, respectively, were classified as having a high-risk waist-hip ratio^27^.

### Other covariates

#### Individual socio-demographic and lifestyle factors

After a thorough review of the literature, the factors that would be controlled in this study were chosen. Age was coded as young old (60–69 years), old-old (70–79 years), and oldest-old (80 + years). Education was categorized as no education/primary schooling not completed, primary completed, secondary completed, and higher and above. Marital status was grouped as currently married, widowed, and others (separated/never married/divorced). Working status was defined as working, retired, and not working. Living arrangement was categorized as living alone, living with a spouse, living with children and living with others. Tobacco and alcohol consumption was coded as no and yes. Physical activity status was coded as frequent (every day), rare (more than once a week, once a week, one to three times in a month), and never. The question through which physical activity was assessed was “How often do you take part in sports or vigorous activities, such as running or jogging, swimming, going to a health centre or gym, cycling, or digging with a spade or shovel, heavy lifting, chopping, farm work, fast bicycling, cycling with loads”?

#### Household/community-related factors

Using household consumption data, the monthly per-capita consumption expenditure (MPCE) quintile was determined. As a summary measure of consumption, the MPCE is computed and utilised and the details of the measurement are explained elsewhere^22^. Further, the variable was split into five quintiles, ranging from the lowest to the richest^22^. Religion was categorized as Hindu, Muslim, Christian, and Others. Caste was recoded as Scheduled Tribe, Scheduled Caste, Other Backward Class (OBC), and others. The Scheduled Castes are a group of people who are socially separated and financially/economically disadvantaged as a result of their low position in the Hindu caste system. The Scheduled Castes and Scheduled Tribes are among India's most economically disadvantaged groups. The OBC refers to a large group of populations who have been classified as "educationally, economically, and socially backward." The OBCs are considered lower castes in the old caste hierarchy system, although the original caste system is not persistent in the country now. The “other” caste group refers to socioeconomically higher population and is regarded to have a better social rank^28^. The place of residence was coded as rural and urban. The region was grouped as North, Central, East, Northeast, West, and South.

### Statistical analysis

The study carried out a bivariate analysis to observe the association between dependent and independent variables. Further, binary logistic regression analysis^29^ was performed to examine the associations after controlling for individual socio-demographic, lifestyle and household/community-related factors. The results were presented in the form of odds ratio (OR) with a 95% confidence interval (CI).

The model is usually put into a more compact form as follows:

$$\mathrm{ln}\left(\frac{{P}_{i}}{1-{P}_{i}}\right)={\beta }_{0}+{\beta }_{1}{x}_{1}+\dots +{\beta }_{M}{x}_{M},$$

where ${\beta }_{0},\dots ,{\beta }_{M}$ are regression coefficient indicating the relative effect of a particular explanatory variable on the outcome. These coefficients change as per the context in the analysis in the study. The study further examined the possible interaction^25,30^ between obese/obesity, high-risk waist circumference, high-risk waist-hip ratio and gender in models 2, 3, and 4. Model 1 (full model) (controlled for all the individual and household characteristics) took into consideration the effect of background characteristics on older adults’ successful ageing rate. In model 2 (controlled for all the individual and household characteristics), the interaction of gender and obesity/overweight among older adults was observed. In model 3 (controlled for all the individual and household characteristics), the study observed the effect of gender and high-risk waist circumference among older adults. Finally, in model 4 (controlled for all the individual and household characteristics), the interaction of gender and high-risk waist-hip ratio among older adults was observed.

### Ethics approval and consent to participate

The data is freely available in the public domain and survey agencies that conducted the field survey for the data collection have collected prior consent from the respondents. The Indian Council of Medical Research (ICMR) and all partner institutions extended the necessary guidance and ethical approval for conducting the LASI survey. Informed consent (verbal and written) was obtained from all subjects and/or their legal guardian(s).

## Results

### Socio-economic and demographic profile of older adults in India (Table 1)

**Table 1:** Socio-economic and demographic profile of older adults in India, 2017–18.

Background characteristics	Men		Women	
	Sample	Percentage	Sample	Percentage
**Obesity-related factors**				
**Obese/overweight**				
No	12,755	84.5	12,568	76.8
Yes	2343	15.5	3798	23.2
**High-risk waist circumference**				
No	13,910	92.1	11,013	67.3
Yes	1188	7.9	5353	32.7
**High-risk waist-hip ratio**				
No	5060	33.5	5038	30.8
Yes	10,038	66.5	11,328	69.2
**Individual factors**				
**Age**				
Young-old	8730	57.8	9678	59.1
Old-old	4702	31.1	4803	29.4
Oldest-old	1666	11.0	1886	11.5
**Education**				
Not educated/primary not completed	8019	53.1	13,314	81.4
Primary	2235	14.8	1297	7.9
Secondary	3096	20.5	1297	7.9
Higher	1748	11.6	458	2.8
**Working status**				
Working	6613	43.8	3108	19.0
Retired	7907	52.4	5593	34.2
Not working	578	3.8	7665	46.8
**Marital status**				
Currently married	12,242	81.1	7211	44.1
Widowed	2489	16.5	8837	54.0
Others	366	2.4	318	2.0
**Living arrangement**				
Living alone	380	2.5	1397	8.5
Living with spouse	3929	26.0	2485	15.2
Living with children and spouse	10,205	67.6	11,268	68.9
Living with others	583	3.9	1216	7.4
**Tobacco consumption**				
No	6197	41.1	12,706	77.6
Yes	8901	59.0	3660	22.4
**Alcohol consumption**				
No	10,939	72.5	15,943	97.4
Yes	4159	27.6	423	2.6
**Physical activity status**				
Frequent	3706	24.6	1966	12.0
Rare	2360	15.6	1672	10.2
Never	9031	59.8	12,729	77.8
**Household factors**				
**MPCE quintile**				
Poorest	3145	20.8	3681	22.5
Poorer	3219	21.3	3611	22.1
Middle	3262	21.6	3331	20.4
Richer	2902	19.2	3136	19.2
Richest	2570	17.0	2607	15.9
**Religion**				
Hindu	12,386	82.0	13,484	82.4
Muslim	1769	11.7	1781	10.9
Christian	388	2.6	511	3.1
Others	555	3.7	590	3.6
**Caste**				
Others	4172	27.6	4556	27.8
Scheduled Caste	2836	18.8	3113	19.0
Scheduled Tribe	1166	7.7	1389	8.5
Other Backward Class	6925	45.9	7308	44.7
**Place of residence**				
Rural	10,879	72.1	11,322	69.2
Urban	4219	28.0	5044	30.8
**Region**				
North	1863	12.3	2096	12.8
Central	3395	22.5	3202	19.6
East	3713	24.6	3729	22.8
Northeast	437	2.9	497	3.0
West	2457	16.3	2941	18.0
South	3233	21.4	3900	23.8
**Total**	15,098	100.0	16,366	100.0

*MPCE:* monthly per capita consumption expenditure.

Overall, 15,098 men (47.5%) and 16,366 women (52.5%) were included in the analysis. It was found that the prevalence of obesity/overweight was higher among older women compared to older men (15.5% vs 23.2%). Similarly, high-risk waist circumference (32.7% vs 7.9%) and high-risk waist-hip ratio (69.2% vs 66.5%) was more prevalent among older women than older men. A higher proportion of older adults belonged to the young-old cohort. The percentage of no education/primary not completed was higher among older women (81.4%) compared to older men counterparts (53.1%). Moreover, the proportion of working older men was higher than working older women (43.8% vs 19.0%). Older women were living more alone compared to older men (8.5% vs 2.5%). Tobacco (59.0% vs 22.4%) and alcohol consumption (27.6% vs 2.6%) was more prevalent among older men than older women counterparts. Older men (24.6%) did more frequent physical activity compared to older women (12%). A higher proportion of older adults were Hindu, belonged to other backward class caste, and lived in rural areas.

### Percentage of older adults with successful ageing outcome by obesity-related measures and socioeconomic and demographic characteristics (Table 2)

**Table 2:** Percentage of older adults with successful ageing outcome by obesity-related measures and socioeconomic and demographic characteristics in India, 2017–2018.

Background characteristics	Men	Women	Differences	p-value
	%	%	%	
**Obesity-related factors**				
**Obese/overweight**				
No	36.5	28.6	7.9	<0.001
Yes	22.7	15.8	6.9	<0.001
**High-risk waist circumference**				
No	37.6	31.6	6.0	<0.001
Yes	17.6	17.2	0.4	0.245
**High-risk waist-hip ratio**				
No	41.0	31.4	9.6	<0.001
Yes	34.2	24.9	9.3	<0.001
**Individual factors**				
**Age**				
Young-old	39.6	30.3	9.3	<0.001
Old-old	29.0	20.6	8.4	<0.001
Oldest-old	21.5	14.2	7.3	<0.001
**Education**				
Not educated/primary not completed	35.2	26.5	8.7	<0.001
Primary	34.9	22.7	12.3	<0.001
Secondary	34.5	21.1	13.4	<0.001
Higher	29.2	21.1	8.2	<0.001
**Working status**				
Working	46.6	37.8	8.8	<0.001
Retired	24.7	22.5	2.2	<0.001
Not working	26.3	23.0	3.3	<0.001
**Marital status**				
Currently married	34.7	30.5	4.2	<0.001
Widowed	31.7	21.6	10.1	<0.001
Others	39.9	25.8	14.1	0.013
**Living arrangement**				
Living alone	33.6	20.4	13.3	<0.001
Living with spouse	28.8	30.2	− 1.4	<0.001
Living with children and spouse	36.7	25.8	10.9	0.076
Living with others	31.6	20.9	10.7	<0.001
**Tobacco consumption**				
No	31.1	25.3	5.8	<0.001
Yes	36.6	26.7	9.9	<0.001
**Alcohol consumption**				
No	34.0	25.3	8.7	<0.001
Yes	35.3	36.4	− 1.2	0.437
**Physical activity status**				
Frequent	44.7	29.3	15.5	<0.001
Rare	44.2	34.1	10.1	<0.001
Never	27.5	23.9	3.6	<0.001
**Household factors**				
**MPCE quintile**				
Poorest	38.8	29.4	9.5	<0.001
Poorer	37.5	27.3	10.2	<0.001
Middle	34.8	27.9	6.9	<0.001
Richer	32.7	22.5	10.2	<0.001
Richest	26.1	18.8	7.3	<0.001
**Religion**				
Hindu	35.3	26.5	8.8	<0.001
Muslim	29.8	18.1	11.7	<0.001
Christian	32.8	29.6	3.2	0.003
Others	28.8	25.4	3.4	<0.001
**Caste**				
Others	31.0	22.3	8.7	<0.001
Scheduled Caste	37.8	24.4	13.4	<0.001
Scheduled Tribe	41.4	40.5	0.8	<0.001
Other Backward Class	33.7	25.4	8.4	<0.001
**Place of residence**				
Rural	37.0	27.7	9.3	<0.001
Urban	27.5	20.8	6.6	<0.001
**Region**				
North	32.7	27.9	4.8	<0.001
Central	42.8	32.1	10.7	<0.001
East	35.4	28.4	7.0	<0.001
Northeast	42.0	31.7	10.3	<0.001
West	27.2	19.7	7.6	<0.001
South	29.5	20.0	9.5	<0.001
**Total**	34.3	25.6	8.7	<0.001

*MPCE:* monthly per capita consumption expenditure.

The study found significant gender differences (men-women: 8.7%) in the prevalence of successful ageing outcomes (p < 0.001). Among the obesity-related factors, the highest gender difference in the successful ageing outcome was witnessed among older adults who were not obese/overweight (p < 0.001), followed by those who had no risk of high waist circumference (p < 0.001) and no risk of waist-hip ratio (p < 0.001). Moreover, among the individual factors, the highest gender difference in the successful ageing outcome was found among those who did the frequent physical activity (p < 0.001), followed by those who had a secondary level of education (p < 0.001) and older adults who lived alone (p < 0.001). Additionally, the maximum gender difference was observed among older adults who belonged to scheduled caste (p < 0.001), followed by Muslim older adults (p < 0.001) and those who belonged to Central India (p < 0.001).

### Estimates from logistic regression analysis for successful ageing among older adults

Table 3 presents four different models. Model 1 shows that the odds of successful ageing were significantly higher among older adults who were not obese/overweight [AOR: 1.31; CI 1.31–1.55], had no high-risk waist circumference [AOR: 1.41; CI 1.29–1.54], and those who had no high-risk waist-hip ratio [AOR: 1.16; CI 1.09–1.24] compared to their counterparts. Interaction results between obese/overweight and sex for successful ageing (Model 2,3 and 4) show that older women who were not obese/overweight had lower odds of successful ageing compared to older men who were not obese/overweight [AOR: 0.86; CI: 0.80–0.93]. Similarly, older women who had no high-risk waist circumference had lower odds of successful ageing compared to older men who had no high-risk waist circumference [AOR: 0.86; CI 0.80–0.96]. Additionally, with reference to older men who had no high risk-hip ratio, older women who had no high risk-hip ratio had lower odds of successful ageing [AOR: 0.81; CI 0.073–89].

**Table 3:** Logistic regression estimates of successful ageing among older adults by obesity-related measures and socioeconomic and demographic characteristics in India, 2017–2018.

Background characteristics	Model-1	Model-2	Model-3	Model-4
	AOR (95%CI)	AOR (95%CI)	AOR (95%CI)	AOR (95%CI)
**Obesity-related factors**				
**Obese/overweight**				
No	1.43* (1.31, 1.55)		1.39* (1.28, 1.51)	1.38* (1.27, 1.51)
Yes	Ref		Ref	
**High-risk waist circumference**				
No	1.41* (1.29, 1.54)	1.42* (1.29, 1.55)		1.43* (1.31, 1.57)
Yes	Ref	Ref		Ref
**High-risk waist-hip ratio**				
No	1.16* (1.09, 1.24)	1.14* (1.09, 1.24)	1.14* (1.09, 1.24)	
Yes	Ref	Ref	Ref	
**Individual factors**				
**Age**				
Young-old	1.75* (1.58, 1.94)	1.75* (1.59, 1.93)	1.75* (1.59, 1.93)	1.75* (1.59, 1.93)
Old-old	1.28* (1.15, 1.43)	1.30* (1.18, 1.44)	1.30* (1.18, 1.44)	1.30* (1.18, 1.44)
Oldest-old	Ref	Ref	Ref	Ref
**Sex**				
Men	1.21* (1.13, 1.30)			
Women	Ref			
**Education**				
Not educated/primary not completed	Ref	Ref	Ref	Ref
Primary	1.01 (0.93, 1.10)	1.00 (0.92, 1.09)	1.00 (0.92, 1.09)	1.00 (0.92, 1.09)
Secondary	1.06 (0.98, 1.16)	1.04 (0.96, 1.13)	1.04 (0.96, 1.13)	1.04 (0.96, 1.13)
Higher	1.06 (0.95, 1.20)	1.00 (0.90, 1.12)	1.00 (0.90, 1.12)	1.00 (0.90, 1.12)
**Working status**				
Working	1.61* (1.48, 1.75)	1.67* (1.54, 1.81)	1.67* (1.54, 1.81)	1.67* (1.54, 1.81)
Retired	0.93* (0.86, 0.98)	0.94 (0.87, 1.01)	0.94 (0.87, 1.01)	0.94 (0.87, 1.01)
Not working	Ref	Ref	Ref	Ref
**Marital status**				
Currently married	1.21* (1.12, 1.3)	1.22* (1.14, 1.31)	1.22* (1.14, 1.31)	1.22* (1.14, 1.31)
Widowed	Ref	Ref	Ref	Ref
Others	1.32* (1.11, 1.58)	1.31* (1.1, 1.55)	1.31* (1.1, 1.55)	1.31* (1.11, 1.55)
**Living arrangement**				
Living alone	1.15 (0.97, 1.37)	1.24* (1.05, 1.47)	1.24* (1.05, 1.47)	1.24* (1.05, 1.46)
Living with spouse	1.22* (1.06, 1.41)	1.12 (0.96, 1.29)	1.12 (0.96, 1.29)	1.12 (0.96, 1.29)
Living with children and spouse	1.23* (1.08, 1.41)	1.21* (1.06, 1.38)	1.21* (1.06, 1.38)	1.21* (1.06, 1.38)
Living with others	Ref	Ref	Ref	Ref
**Tobacco consumption**				
No	1.03 (0.97, 1.1)	1.01 (0.95, 1.07)	1.01 (0.95, 1.07)	1.00 (0.95, 1.07)
Yes	Ref	Ref	Ref	Ref
**Alcohol consumption**				
No	1.09* (1.01, 1.17)	1.09* (1.01, 1.18)	1.09* (1.01, 1.18)	1.09* (1.01, 1.18)
Yes	Ref	Ref	Ref	Ref
**Physical activity status**				
Frequent	1.31* (1.22, 1.41)	1.34* (1.25, 1.44)	1.34* (1.25, 1.44)	1.34* (1.25, 1.44)
Rare	1.26* (1.16, 1.36)	1.29* (1.19, 1.39)	1.29* (1.19, 1.39)	1.29* (1.19, 1.39)
Never	Ref	Ref	Ref	Ref
**Household factors**				
**MPCE quintile**				
Poorest	Ref	Ref	Ref	Ref
Poorer	0.91* (0.84, 0.99)	0.93 (0.86, 1.00)	0.93 (0.86, 1.00)	0.93 (0.86, 1.00)
Middle	0.87* (0.8, 0.95)	0.90* (0.83, 0.97)	0.90* (0.83, 0.97)	0.90* (0.83, 0.97)
Richer	0.76* (0.7, 0.83)	0.78* (0.72, 0.84)	0.78* (0.72, 0.84)	0.78* (0.72, 0.85)
Richest	0.67* (0.61, 0.73)	0.68* (0.62, 0.74)	0.68* (0.62, 0.74)	0.68* (0.62, 0.74)
**Religion**				
Hindu				
Muslim	0.77* (0.7, 0.85)	0.77* (0.71, 0.84)	0.77* (0.71, 0.84)	0.77* (0.71, 0.84)
Christian	1.06 (0.94, 1.18)	1.06 (0.95, 1.17)	1.06 (0.95, 1.17)	1.06 (0.95, 1.17)
Others	0.93 (0.81, 1.06)	0.93 (0.82, 1.06)	0.93 (0.82, 1.06)	0.93 (0.82, 1.06)
**Caste**				
Others	Ref	Ref	Ref	Ref
Scheduled Caste	1.00 (0.92, 1.10)	0.99 (0.91, 1.08)	0.99 (0.91, 1.08)	0.99 (0.91, 1.08)
Scheduled Tribe	1.36* (1.24, 1.50)	1.38* (1.25, 1.51)	1.38* (1.25, 1.51)	1.37* (1.25, 1.5)
Other Backward Class	1.04 (0.97, 1.12)	1.04 (0.97, 1.12)	1.04 (0.97, 1.12)	1.04 (0.97, 1.12)
**Place of residence**				
Rural	Ref	Ref	Ref	Ref
Urban	0.96 (0.9, 1.02)	0.93* (0.87, 0.98)	0.93* (0.87, 0.98)	0.93* (0.87, 0.99)
**Region**				
North	Ref	Ref	Ref	Ref
Central	1.18* (1.07, 1.30)	1.20* (1.10, 1.32)	1.2* (1.1, 1.32)	1.20* (1.1, 1.32)
East	0.92 (0.84, 1.01)	0.92 (0.84, 1.00)	0.92 (0.84, 1.00)	0.92 (0.84, 1.00)
Northeast	1.35* (1.21, 1.51)	1.33* (1.19, 1.48)	1.33* (1.19, 1.48)	1.32* (1.19, 1.47)
West	0.69* (0.62, 0.76)	0.69* (0.63, 0.76)	0.69* (0.63, 0.76)	0.69* (0.63, 0.76)
South	0.73* (0.66, 0.8)	0.72* (0.66, 0.79)	0.72* (0.66, 0.79)	0.72* (0.66, 0.79)
**Obese/overweight # sex**				
No # men		Ref		
No # women		0.86* (0.80, 0.93)		
Yes # men		0.70* (0.63, 0.77)		
Yes # women		0.64* (0.56, 0.73)		
**High-risk waist circumference # sex**				
No # men			Ref	
No # women			0.86* (0.80, 0.93)	
Yes # men			0.66* (0.56, 0.77)	
Yes # women			0.63* (0.57, 0.69)	
**High-risk waist-hip ratio # sex**				
No # men				Ref
No # women				0.81* (0.73, 0.89)
Yes # men				1.00 (0.92, 1.08)
Yes # women				0.90* (0.82, 0.99)

Model-1, 2, 3 and 4 was controlled for all individual and household characteristics.

*Ref:* reference, #: interaction, *MPCE:* monthly per capita consumption expenditure, *AOR:* adjusted odds ratio, *CI:* confidence interval.

*if p < 0.05.

## Discussion

In this cross-sectional study of nearly 31,500 older Indian men and women, we found more than 34% of older men and nearly 26% of older women in our study sample qualified as successful agers. The rate of successful ageing in our study is substantially higher compared to a recent study conducted in China using similar components, with around 19% of older men and 9.5% of older women being successful agers^8^. Possible reasons for higher rate of successful aging in India than China may be related to the use of different assessment tools for components of depression and cognition, eg., the Chinese study considered the Centre for Epidemiological Study Depression (CESD) scale for measuring depressive symptoms, whereas we used CIDI-SF scale that measures major depressive disorder. Another explanation is the lack of awareness and under-diagnosis of chronic diseases among older adults in India than in China. However, the rate is a little lower in comparison to an earlier review of 29 larger quantitative studies by Depp et al.^12^ that reported more than one-third of the community-dwelling older individuals (35.8%) on average qualified as successful agers. The review concluded that the nature of definitions and selected domains can lead to considerable variation in the proportion of successfully aging population.

The female disadvantage observed in successful ageing in this study may be explained by the gender paradox in health where, women with worse health tend to live longer than men^31^, and it is consistent with previous studies in other developing countries^32,33^. These differences seem to be attributed to men having improved cognition, increased physical function and social engagement than women in India^34–37^. In addition, it is documented that compared to men, women experience greater barriers to health service use, especially among socioeconomically poor people in India^38^. Furthermore, younger age and being married was associated with successful aging in comparison to old-old and widowed counterparts in this study, and the finding are consistent with previous studies^39–42^. The negative association between increasing age and achieving successful aging is expected due to the increase in functional, cognitive and biological decline with ageing. Besides, older adults who lived with a spouse or with children and spouse were significantly more likely to achieve successful ageing compared to those who lived with others in this study. This is in line with the argument that unlike Western model of successful aging, older individuals in India and other Asian countries focus more on familism and value the roles of spouse and children in successful aging^43^.

In the present study, we observed that obesity/ overweight, high-risk waist circumference and waste-hip ratio to be associated with a substantially reduced likelihood of successful ageing. This could be attributed to the increased lifestyle diseases associated with obesity and other adverse anthropometric measures^32^. These findings are also in parallel with studies that have documented nutrition and related anthropometric indices playing a pivotal and crucial role in maintaining older individuals’ physical capacity and optimal health^44–46^. Similarly, factors such as healthy nutrition, increased physical activity, improved stress management and greater resilience were found to be predictors of successful ageing^32,47–49^. Another study suggests that since most older individuals are more susceptible to foodborne illness, nutrition-based services should be provided to ensure successful ageing^50^. Further, physically active individuals in this study were more likely to have successful ageing than those who never did physical activity, which concurs with previous studies that suggest that physical activity reduces the progression of chronic disease and disabling conditions in older people and increase active life expectancy^49,51,52^. Similarly, compared to not working older adults, working older adults were more likely to have successful ageing and this finding supports the fact that older adults in the workforce maintain the same level of activity and social relationships as in earlier life stages and develop proactive behaviors for successful aging at work^53,54^.

To further determine whether high-risk status in obesity-related measures has different associations with successful ageing between men and women, we also tested for interaction between measures related to obesity and sex of the older individuals. In terms of women, compared with the older men who were normal weight, the obese women older population was significantly less likely to have successful ageing. This was similar with regards to high-risk waist circumference and waste-hip ratio. To illustrate further, the differences between older men and women in the rate of successful ageing with women being highly unsuccessful can be related to multiple factors. Firstly, the higher prevalence of obesity in women is a risk factor for arthritis and chronic conditions^55–57^. On the other hand, the higher obesity risk of women could be linked to their lower physical activity compared with men, particularly in societies with particular gender norms such as cultural inappropriateness of exercise by women and adverse environment with no or minimum accessible recreational areas/facilities^58^, which discourage physical activity in women throughout their life^59,60^.

The current study was subjected to several limitations. The observed gender disparities might have occurred due to misclassification in reporting symptoms because studies suggest that women are more likely to report depression and disability than men. Again, chronic diseases in our study are self-reported, leading to several biases and eventually influencing the rate of successful ageing; therefore, future research should look at the unsuccessful ageing caused by specific components rather than just looking at a composite index score. Further, importantly, the cross-sectional study cannot conclude causal inference between obesity-related measures and successful ageing; the follow-up in the future can, however, support the persuaded evidence. Nevertheless, the main strengths of this study include the conceptualization of successful ageing based on multiple outcomes, including validated measures of depression and cognitive functioning and objective measures of functioning. The large sample size and multiple covariates, which appear to be confounders, along with comprehensive information on socioeconomic characteristics of the ageing population, are further advantages.

## Conclusion

Being overweight/ obese and having high-risk waist circumference and waist-hip ratio were found to be significant factors associated with less successful aging among older adults, especially women in this study. The current findings highlight the importance of understanding the modifiable factors, including nutritional awareness and developing targeted strategies for promoting successful ageing. The modifiable risk factors such as obesity and physical inactivity are obvious candidates for health promotion efforts in which health decision-makers can invest through supporting healthy eating and exercise among older people. Moreover, since Asian countries including India has had a relatively short history of tackling the issues related to ageing unlike developed countries, and for other reason, research into successful ageing has been lacking in many of these countries^61^, suggesting that further investigation is required with more follow-up survey information.

## Data Availability

The study utilizes a secondary source of data that is freely available in the public domain through a request from https://iipsindia.ac.in/sites/default/files/LASI_DataRequestForm_0.pdf.

## Abbreviations

AOR: Adjusted odds ratio
CI: Confidence interval
ADL: Activities of daily living
CIDI-SF: Short form composite international diagnostic interview
MPCE: Monthly per capita consumption expenditure
OBC: Other backward class
SC: Scheduled castes
ST: Scheduled tribes
